# Microdamage Caused by Fatigue Loading in Human Cancellous Bone: Relationship to Reductions in Bone Biomechanical Performance

**DOI:** 10.1371/journal.pone.0083662

**Published:** 2013-12-30

**Authors:** Floor M. Lambers, Amanda R. Bouman, Clare M. Rimnac, Christopher J. Hernandez

**Affiliations:** 1 Sibley School of Mechanical and Aerospace Engineering, Cornell University, Ithaca, New York, United States of America; 2 Department of Biomedical Engineering, Cornell University, Ithaca, New York, United States of America; 3 Department of Mechanical and Aerospace Engineering, Case Western Reserve University, Cleveland, Ohio, United States of America; 4 Hospital for Special Surgery, New York, New York, United States of America; University of Notre Dame, United States of America

## Abstract

Vertebral fractures associated with osteoporosis are often the result of tissue damage accumulated over time. Microscopic tissue damage (microdamage) generated *in vivo* is believed to be a mechanically relevant aspect of bone quality that may contribute to fracture risk. Although the presence of microdamage in bone tissue has been documented, the relationship between loading, microdamage accumulation and mechanical failure is not well understood. The aim of the current study was to determine how microdamage accumulates in human vertebral cancellous bone subjected to cyclic fatigue loading. Cancellous bone cores (n = 32) from the third lumbar vertebra of 16 donors (10 male, 6 female, age 76±8.8, mean ± SD) were subjected to compressive cyclic loading at σ/E_0_ = 0.0035 (where σ is stress and E_0_ is the initial Young’s modulus). Cyclic loading was suspended before failure at one of seven different amounts of loading and specimens were stained for microdamage using lead uranyl acetate. Damage volume fraction (DV/BV) varied from 0.8±0.5% (no loading) to 3.4±2.1% (fatigue-loaded to complete failure) and was linearly related to the reductions in Young’s modulus caused by fatigue loading (r^2^ = 0.60, p<0.01). The relationship between reductions in Young’s modulus and proportion of fatigue life was nonlinear and suggests that most microdamage generation occurs late in fatigue loading, during the tertiary phase. Our results indicate that human vertebral cancellous bone tissue with a DV/BV of 1.5% is expected to have, on average, a Young’s modulus 31% lower than the same tissue without microdamage and is able to withstand 92% fewer cycles before failure than the same tissue without microdamage. Hence, even small amounts of microscopic tissue damage in human vertebral cancellous bone may have large effects on subsequent biomechanical performance.

## Introduction

Vertebral fractures are the most common form of osteoporosis-related fractures [1], [2]. Only 51% of all vertebral fractures are associated with a discrete loading event, suggesting that many vertebral fractures are the result of tissue damage caused by multiple loading events over time [3], [4]. Cancellous bone is the primary load-carrying component in human vertebral bodies, suggesting that damage accumulation and related degradation in biomechanical performance of vertebral cancellous bone is part of the development of vertebral fractures [5].

Microscopic tissue damage (microdamage) in the form of linear microcracks, diffuse damage, or trabecular microfracture occurs *in vivo* [6], [7], shows a higher prevalence in older individuals [8], [9], [10], and is believed to be a biomechanically relevant aspect of bone quality [11]. Microdamage in bone tissue can be visualized through bulk staining with basic fuchsin [12] or fluorochromes [13], [14] followed by sectioning and histomorphometry. Alternatively microdamage may be stained using radio-opaque contrast agents combined with x-ray computed tomography (micro-CT, etc) [15], [16], [17]. Previous studies have demonstrated that greater amounts of cyclic loading applied to cortical bone tissue are related to greater amounts of microdamage [18], [19], [20], [21], [22], [23]. In cancellous bone, relations between a single loading event and the amount of resulting microdamage have been reported [16], [24], [25], [26], but less is known about microdamage accumulation during cyclic fatigue loading.

**Figure 1:**
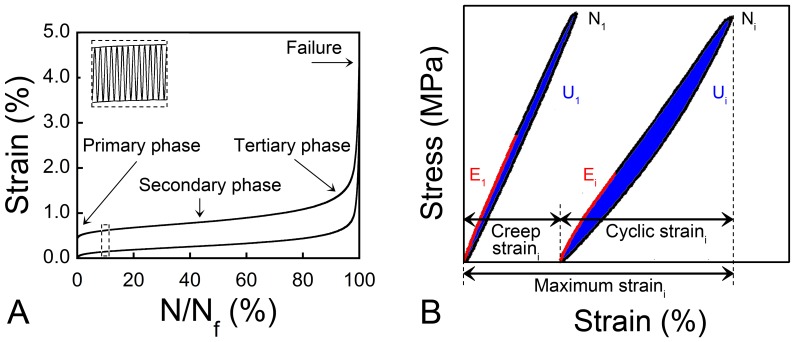
Fatigue loading of cancellous bone. (A) A creep-fatigue curve is shown. Lines represent minimum and maximum strain per cycle, as indicated in the inset. The creep-fatigue curve consists of three phases: the primary phase where the strain accumulation per cycle decreases, the secondary phase where the strain accumulation per cycle is constant, and the tertiary phase where the strain accumulation per cycle increases. (B) Stress-strain plots for the first load cycle (N_1_) and a later load cycle (N_i_) are shown. Mechanical properties measured within the loading cycle include the Young’s modulus (E, shown in red), energy dissipation (U, measured as the area labeled in blue), maximum strain_i_ = creep strain_i_+cyclic strain_i_.

Cancellous bone tissue submitted to cyclic loading displays creep-fatigue behavior in three phases: a primary phase in which stiffness reduces relatively rapidly, a secondary phase in which stiffness declines slowly and a tertiary phase in which stiffness rapidly declines before failure (**Fig. 1**) [27], [28], [29]. Microdamage generated during fatigue loading has been examined in bovine cancellous bone [13], [30], [31] and in human femoral cancellous bone after an initial overload [32], but quantitative relationships between the amount of fatigue loading, microdamage and alterations in biomechanical performance in less dense cancellous bone such as that in the vertebral body have not yet been presented. Human vertebral cancellous bone is more rod-like and porous than cancellous bone tissue from the femoral neck and is therefore expected to show different failure processes [33].

The overall goal of the current line of investigation is to understand the contributions of microdamage to vertebral fractures. Specifically, the aim of the current study is to determine the relationship between the amount of fatigue loading, the accumulation of microdamage, and reductions in the Young’s modulus during cyclic loading of human vertebral cancellous bone.

## Methods

### Sample Preparation

The third lumbar vertebral bodies of 16 donors (10 male, 6 female, mean age 76±8.8 years, range 62–92 years) were examined (tissue from NDRI, Philadelphia, PA). The work examined human tissue acquired from a licensed tissue bank (http://ndriresource.org/) and was therefore exempt from Institutional Review Board evaluation. Donors had no medical history of metabolic bone disease or cancer. Vertebrae were dissected free of soft tissue and cylindrical cores of cancellous bone (nominally 8 mm diameter, 19 mm in length) aligned in the superior-inferior direction (as described previously [34]) were collected using a diamond tipped coring tool (Starlite Industries, Bryn Mawr, PA, USA). Trabecular alignment was ensured by aligning the vertebrae visually during specimen collection. Two specimens were collected from each vertebra resulting in a total of 32 specimens. Specimens from the anterior, left, or right sites in the vertebra were included in the study. Little variation in mechanical properties has been observed among anterior, left and right sites within lumbar vertebrae [35], [36], [37]. Samples from the posterior side were avoided because of bone volume fraction inhomogeneity due to the vascular supply. Specimens were wrapped in saline soaked gauze and stored in airtight containers at −20°C until mechanical testing. Prior to mechanical testing, bone marrow was removed from the specimens with a low-pressure water jet. The specimens were glued (Loctite 401, Newington, CT, USA) into brass cylindrical end-caps as previously described [34]. The cyanoacrylate glue was allowed to cure overnight at 4°C, with specimens wrapped in saline soaked gauze to maintain hydration.

### Mechanical Loading

Fatigue loading was applied to the cancellous bone cores to induce microdamage. Mechanical loading was conducted at room temperature (23°C) using a servohydraulic testing machine (858 Mini Bionix, MTS, Eden Prairie, MN, USA) with a 1 kN load cell (Model 661.18E-01, MTS, Eden Prairie, MN, USA, USA). Mechanical properties degrade faster at higher temperatures [28], [38], [39], thus testing at body temperature would have accelerated mechanical degradation compared to testing at room temperature. By testing at room temperature in the current study, microdamage propagated more slowly and we were better able to stop fatigue loading at prescribed amounts of fatigue loading. Strain was measured with a 25 mm gage length extensometer attached to the end-caps. The effective gage length was defined as the exposed plus half the embedded length of the specimen [34]. During testing, specimens were hydrated with physiologically buffered saline (pH of 7.4) containing 10 µM protease inhibitors (E-64, Sigma Aldrich, St. Louis, MO, USA). A rubber membrane placed around the specimen was used to contain the solution. Mechanical loading consisted of an initial set of ten preconditioning cycles between 0 and 0.1% strain at a rate of 0.5%/sec. After preconditioning, the specimens were submitted to cyclic loading between 0 N and a load corresponding to a value of σ/E_0_ of 0.0035 mm/mm, where σ is stress and E_0_ is the initial Young’s modulus. Fatigue loading was performed at 4 Hz in load control using a haversine waveform.

**Figure 2:**
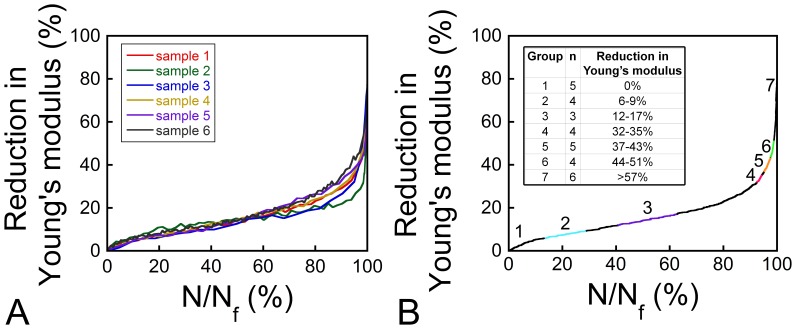
Reduction in Young’s modulus with fatigue loading. (A) Reduction in Young’s modulus for 6 samples loaded to failure is shown. Each color represents a different specimen. (B) The average reduction in Young’s modulus of all samples loaded to failure is shown relative to the proportion of fatigue life used. The numbers and colored ranges on the curve indicate the points where fatigue loading was stopped for each group (the range shown in the inset).

Six specimens were loaded to 5% strain, which we defined as failure (group 7). The value of 5% strain was defined as failure, because from preliminary tests it was found that loading to 5% strain would capture the primary, secondary, and tertiary phase. Our definition is conservative in that others have defined failure at lower strain magnitudes (2.5% [30] or even “the cycle before which a specimen could no longer sustain the applied stress” which was at a strain of 3.36% ±2.13% [29]). Five specimens were not subjected to fatigue loading (group 1). Fatigue loading in five other groups was stopped before failure based on monitoring the creep-fatigue curve, secant modulus, and hysteresis loops to achieve specimens loaded to a specified region of the creep-fatigue curve. Fatigue loading was stopped at the start of the secondary phase (group 2), in the middle of the secondary phase (group 3), and at three different points within the tertiary phase (groups 4–6). Specimen group assignment was performed to evenly distribute male and female donors and ensure that multiple specimens from the same donor were not in the same group. After loading, the stress-strain curves were examined to confirm group classification based on reduction in Young’s modulus (1 − E/E_0_), and specimens were reassigned group number if necessary. Eleven specimens were reassigned group based on the Young’s modulus reduction. Reassignment did not alter the relatively even distribution of male/female donors and specimens among donors. The distribution in Young’s modulus reduction is shown in Figure 2B (inset): For group 1 (n = 5) no fatigue loading was applied, for the other groups the Young’s modulus reduction was 6–9% (group 2, n = 4), 12–17% (group 3, n = 3), 32–35% (group 4, n = 4), 37–43% (group 5, n = 5), 44–51% (group 6, n = 4) or 57–82% (group 7, n = 6).

### Mechanical Properties

Force and strain data were collected every cycle at 102.4 Hz, and at 1024 Hz every tenth cycle and during the last 10 cycles (MTS TestSuite Multipurpose Elite/FlexTest 40 digital controller, MTS, Eden Prairie, MN, USA). Young’s modulus was calculated from the data sampled at 1024 Hz. Young’s modulus (E) was determined with a polynomial fit of the first 0.2% portion of the loading segment [40]. Force data was converted to stress using the cross-sectional area of the specimen gage region. Creep strain, cyclic strain, and maximum strain were determined per cycle (**Fig. 1B**). Creep strain was defined as the minimum strain and cyclic strain as the difference between the maximum and minimum strain per cycle [41], [42]. Maximum strain was the sum of the creep and cyclic strain [28], [29]. Energy dissipation (U), which describes the amount of energy lost per cycle, was calculated as the area within the hysteresis loop of each cycle. The number of cycles to failure (N_f_) was determined for specimens loaded to failure (defined as achieving a maximum strain of 5%).

### Microdamage and Bone Microarchitecture

After mechanical testing, specimens were removed from the end-caps using a low-speed diamond saw (Isomet, Buehler Ltd., Lake Bluff, IL) and stained with lead uranyl acetate. Lead uranyl acetate binds to regions of bone tissue in which microdamage has formed [43] and has high x-ray absorption, allowing visualization of microdamage with micro-computed tomography [16], [17]. Lead uranyl acetate staining was performed as follows (derived from [16]): cylindrical cores were dehydrated in 70% acetone for 24 hours. Next, the specimens were stained for one week in a solution of equal parts 8% uranyl acetate (Ted Pella, Inc., Redding, CA, USA ) in 70% acetone and 20% lead (II) acetate (Sigma-Aldrich, St. Louis, MO, USA) in 70% acetone, while on a shaker covered to limit exposure to light. Thereafter, the samples were placed in a 1% ammonium sulfide (Sigma-Aldrich, St. Louis, MO, USA) in 70% acetone solution for one week with one change of solution at 3 days. Samples were rinsed with 70% acetone, submersed in 70% acetone for one week, rinsed again and submitted to ultrasonic vibration for 10 minutes in 70% acetone to remove the solution from the pores. Specimens were glued on metal nails. Images were collected with X-ray microscopy (VersaXRM-500, Xradia, Pleasanton, CA, USA) in air at an energy of 100 kVp, a current of 90 µA, an integration time of 1000 ms, and an isotropic voxel size of 10 µm. The region of interest was 10.2 mm in length and contained the complete cross-sections of the specimen. The volume analyzed was 7.5 mm in diameter (at least 250 µm away from machined edges) and 8.2 mm in length (central slices), unless the specimen was shorter, in which case the volume analyzed started 500 µm below the machined edge. To segment bone, a Gaussian filter (sigma = 1.2, support = 1) was applied to the images, followed by a global threshold, determined with the Otsu method. To segment microdamage from bone, manual thresholds were chosen by an observer blinded to the experimental group of each specimen. Bone microarchitecture was quantified using a three-dimensional approach (IPL, Scanco Medical, Brüttisellen, Switzerland), including bone volume fraction (BV/TV), trabecular thickness, trabecular number, bone surface, structure model index, connectivity density, and degree of anisotropy. Damage volume fraction was calculated as the damage volume divided by the bone volume (DV/BV).

### Statistical Analysis

Regression was performed to determine the relationship between loading group (as an ordinal) and mechanical properties or DV/BV. Regression models were also performed to determine relations between microdamage, mechanical properties and measures of cancellous microarchitecture. Donor was included as a random effect to take into account the use of multiple specimens from each donor. Statistical tests were conducted using JMP 9 (SAS Institute Inc., Cary, NC, USA) or MATLAB (7.12.1, R2011a, The MathWorks Inc., Natick, MA, USA).

## Results

### Damage Volume Fraction

**Figure 3:**
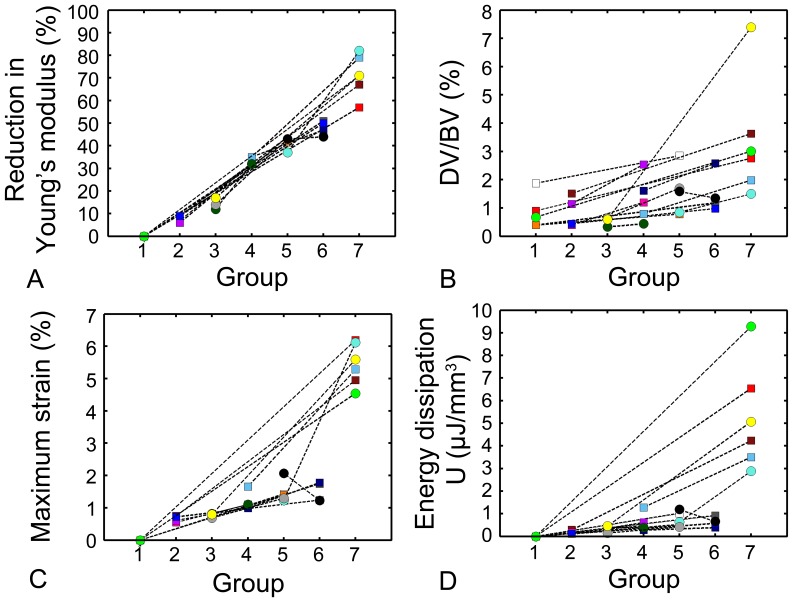
The distribution of DV/BV and mechanical properties for each of the groups. Colors represent different donors. Female donors are shown as circles, male donors as squares. Lines connect specimens from the same donor. (A) Reduction in Young’s modulus (r^2^ = 0.96, p<0.01), (B) Damage volume fraction (r^2^ = 0.71, p<0.01), (C) Maximum strain (r^2^ = 0.93, p<0.01), and (D) Maximum energy dissipation (r^2^ = 0.65, p<0.01) were increased in groups experiencing more fatigue loading.

**Figure 4:**
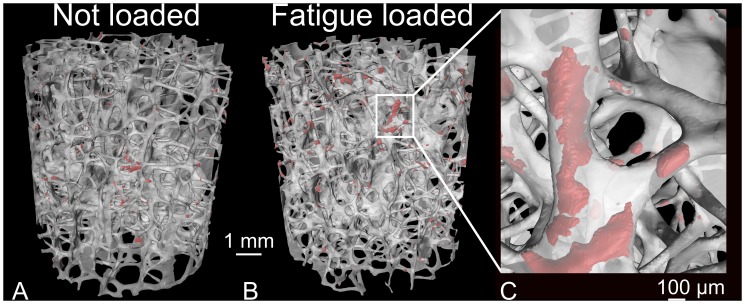
Visualization of microdamage in cancellous bone. Red represents microdamage and transparent white represents bone. Shown are two specimens from the same donor subjected to (A) No loading (Group 1) and (B) Fatigue loading in group 5 (Young’s modulus was reduced by 42%). (C) An enlarged view of a damage site in the cancellous bone is shown.

Damage volume fraction was increased in specimens receiving more fatigue loading (r^2^ = 0.71, p<0.01, **Fig. 3B**). DV/BV was 0.8% ±0.6% (mean±SD) in samples with no fatigue loading and 3.4% ±2.1% in the group loaded to failure. Although there was considerable variability within each loading group, within a donor, DV/BV was greater in specimens with greater amounts of fatigue loading (visualization of specimens from the same donor in **Fig. 4**).

### Mechanical Properties

**Figure 5:**
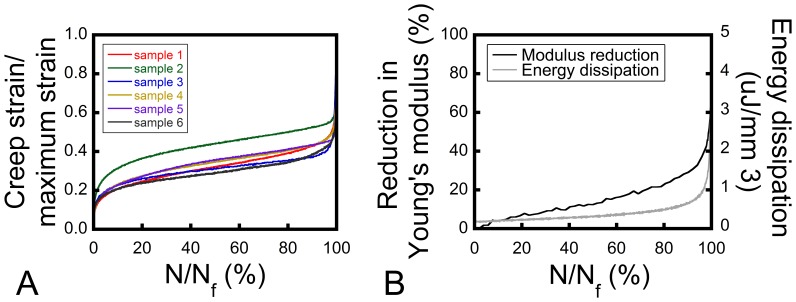
Changes in mechanical properties with fatigue loading. (A) The proportion of creep strain to maximum strain is shown for the six specimens fatigue loaded to failure. Each color represents a different specimen. (B) The alterations in modulus and energy dissipation in one specimen fatigue loaded to failure is shown. Modulus reduction increased progressively throughout fatigue loading, with a rapid loss in stiffness close to failure. Energy dissipation remained relatively constant until in the tertiary phase, where it increased rapidly.

Young’s modulus was reduced in specimens receiving more fatigue loading (r^2^ = 0.96, p<0.01, **Fig. 3A**). Maximum strain was increased in specimens receiving more fatigue loading, but did not exceed 2% in the groups where cyclic loading was stopped before failure (Groups 1–6, **Fig. 3C**). Maximum energy dissipation ranged between 0.1 µJ/mm^3^ and 1.3 µJ/mm^3^ for groups 2–6, and increased to an average of 4.6 µJ/mm^3^ for group 7 (r^2^ = 0.65, p<0.01, **Fig. 3D**). Early during fatigue loading the maximum strain was similar to the cyclic strain, but with continued loading, creep strain increased (**Fig. 5A**), indicating that changes in maximum strain were mostly an effect of accumulation of creep strain. Maximum strain was related to both creep strain (r^2^ = 0.95, p<0.01) and cyclic strain (r^2^ = 0.64, p<0.01, **Table 1**). In contrast to the continuous reduction in Young’s modulus throughout fatigue loading, energy dissipation remained relatively constant and only increased in the tertiary phase (**Fig. 5B**).

**Table 1:** Regression models relating measured mechanical properties and DV/BV are shown.

Independent variable	Dependent variable	r^2^	Slope (95% CI)	Intercept (95% CI)
**Reduction in Young’s** **Modulus (%)**	DVBV (%)	0.60	0.034 (0.018,0.050)	0.434 (−0.265,1.134)
**Maximum strain (%)**	DVBV (%)	0.61	0.491 (0.283,0.699)	0.690 (0.125,1.255)
**Creep strain (%)**	DVBV (%)	0.57	0.596 (0.327,0.864)	0.905 (0.377,1.433)
**Cyclic strain (%)**	DVBV (%)	0.54	2.029 (1.094,2.964)	0.200 (−0.550,0.950)
**Energy dissipation (µJ/mm^3^)**	DVBV (%)	0.53	0.413 (0.221,0.605)	1.014 (0.512,1.516)
**Maximum strain (%)**	Reduction in Young’sModulus (%)	0.60	11.18 (8.54,13.82)	13.27 (7.07,19.48)
**Creep strain (%)**	Reduction in Young’sModulus (%)	0.35	12.86 (9.10,16.62)	18.93 (12.80,25.05)
**Cyclic strain (%)**	Reduction in Young’sModulus (%)	0.83	53.87 (45.58,62.15)	−3.06 (−9.37,3.26)
**Energy dissipation (µJ/mm^3^)**	Reduction in Young’sModulus (%)	0.46	8.05 (4.71,11.39)	22.44 (14.34,30.55)
**Creep strain (%)**	Maximum strain (%)	0.95	1.22 (1.17,1.28)	0.42 (0.33,0.52)
**Cyclic strain (%)**	Maximum strain (%)	0.64	4.01 (3.22,4.80)	−0.91 (−1.51, −0.33)
**Energy dissipation (µJ/mm^3^)**	Maximum strain (%)	0.76	0.75 (0.57,0.93)	0.77 (0.32,1.24)

Donor was included as a random effect in all of the regression models to account for the use of multiple specimens from each donor.

### Relationship between Damage Volume Fraction and Mechanical Properties

**Figure 6:**
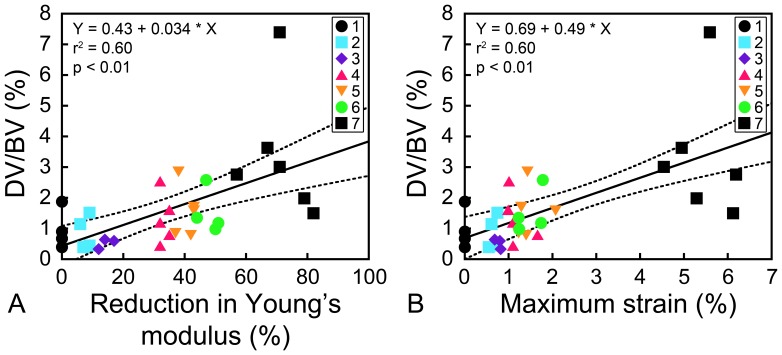
The relationships between DV/BV and mechanical properties. A positive relation between DV/BV and (A) reduction in Young’s modulus (r^2^ = 0.60, p<0.01); and (B) maximum strain applied during cyclic loading (r^2^ = 0.61, p<0.01) was observed. Dotted lines indicate the 95% confidence interval of the regression line.

Greater amounts of microdamage were associated with reductions in mechanical properties (**Fig. 6**). DV/BV was positively related to modulus reduction (r^2^ = 0.60, p<0.01) and maximum strain (r^2^ = 0.61, p<0.01). Creep strain (r^2^ = 0.57, p<0.01), cyclic strain (R^2^ = 0.54, p<0.01), and energy dissipation (r^2^ = 0.53, p<0.01) were positively correlated with DV/BV (**Table 1** **,** **Table 2** **, Fig. S1**).

**Table 2:** Correlations between mechanical properties, DV/BV and parameters of bone microarchitecture are shown.

	Reduction inYoung’s modulus (%)	Maximumstrain (%)	Creepstrain (%)	Cyclicstrain (%)	Energydissipation (µJ/mm^3^)	DV/BV (%)	TrabecularThickness (um)	BV/TV (%)
**Reduction in Young’s modulus (%)**	–	(0.74,0.93)	(0.66,0.91)	(0.87,0.97)	(0.46,0.84)	(0.30,0.78)	(−0.34,0.35)	(−0.50,0.17)
**Maximum strain (%)**	0.87 *	–	(0.98,1.0)	(0.79,0.95)	(0.71,0.92)	(0.39,0.82)	(−0.25,0.44)	(−0.40,0.29)
**Creep strain (%)**	0.82 *	0.99 *	–	(0.68,0.91)	(0.67,0.91)	(0.37,0.80)	(−0.25,0.44)	(−0.37,0.33)
**Cyclic strain (%)**	0.94 *	0.9 *	0.83 *	–	(0.66,0.91)	(0.36,0.80)	(−0.28,0.42)	(−0.52,0.15)
**Energy dissipation (µJ/mm^3^)**	0.70 *	0.85 *	0.83 *	0.82 *	–	(0.36,0.80)	(−0.20,0.48)	(−0.33,0.36)
**DV/BV (%)**	0.59 *	0.65 *	0.63 *	0.63 *	0.63 *	–	(−0.25,0.44)	(−0.37,0.33)
**Trabecular Thickness (um)**	−0.01	0.11	0.11	0.08	0.16	0.1	–	(−0.46,0.22)
**BV/TV (%)**	−0.19	−0.06	−0.02	−0.21	0.02	−0.02	−0.14	–

The lower triangle displays the correlation coefficients and the 95% confidence interval of the correlation coefficient is shown in the upper triangle. Correlation scatterplots are included in Fig. S1.

p<0.05.

### Relationship between Damage Volume Fraction and Fatigue Life

**Figure 7:**
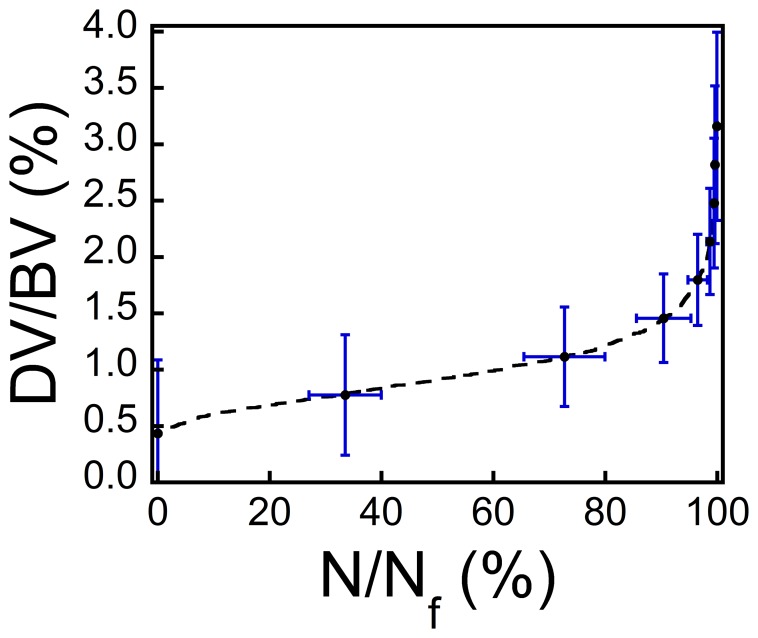
The estimated relationship between the amount of microdamage (DV/BV) and the proportion of fatigue life used (N/N_f_). The relationship is derived from the average reduction in Young’s modulus in Fig. 2B and regression model in Fig. 6A. Error bars in blue indicate the 95% confidence intervals at specified points.

The relationship between reductions in Young’s modulus and proportion of fatigue life was consistent among specimens loaded to failure (n = 6, **Fig. 2A**) allowing quantitative estimates of the relationship between DV/BV and proportion of fatigue life by combining the relationship between the reduction in Young’s modulus and proportion of fatigue life (**Fig. 2B**) with the relationship between the reduction in Young’s modulus and DV/BV (**Fig. 6A**). The relationship between DV/BV and the proportion of fatigue life was nonlinear, with most damage accumulation late in the fatigue life (**Fig. 7**). A DV/BV of 1.5% (95% CI: 1.1–1.9%) corresponded, on average, to a 31% reduction in Young’s modulus and to 92% (95% CI: 88–96%) of the fatigue life. A DV/BV of 2% was associated, on average, with 98% of the fatigue life.

### Effect of Cancellous Microarchitecture on Microdamage Accumulation

No differences in bone microarchitectural parameters (**Table 3**) were detected among groups. No correlations between DV/BV and microarchitecture were observed (**Table 2** **, Fig. S1**). Including microarchitectural parameters as a covariate in the regression between DV/BV and mechanical properties did not improve the correlation coefficients.

**Table 3:** The mean and standard deviation of the bone microarchitectural parameters are shown.

	Mean±SD	Range
**Bone volume fraction (%)**	8.47±2.05	5.53–15.04
**Trabecular thickness (µm)**	128±13	106–149
**Trabecular number (1/mm)**	0.88±0.15	0.60–1.28
**Bone surface (mm^2^)**	631±154	371–1104
**Structure model index (−)**	1.51±0.32	0.64–2.07
**Degree of anisotropy (−)**	1.55±0.21	1.28–2.16
**Connectivity density (1/mm^3^)**	3.06±1.23	1.00–6.68

## Discussion

The current study provides the first quantitative measures of stained microdamage accumulation during cyclic loading in human vertebral cancellous bone and shows that microdamage generated by cyclic loading is linearly related to reductions in specimen stiffness and non-linearly related to the proportion of fatigue life. Additionally, our results suggest that microdamage has a greater effect on fatigue life of cancellous bone tissue than it does on Young’s modulus.

There are a number of strengths to the current study. First, our study describes microdamage accumulation with cyclic loading both in terms of microscopically observed microdamage as well as reductions in specimen stiffness. Second, cyclic loading was applied to vertebral cancellous bone from elderly humans with low bone volume fraction (BV/TV range 5–15%), thereby characterizing the population in which vertebral fractures are more prevalent. Third, microdamage was quantified in three-dimensions. Examination of microdamage in three-dimensions reduces variance in microdamage measures by measuring the entire specimen [44].

There are some limitations that must be considered in interpreting our results. First, the current study measured microdamage based on lead uranyl acetate staining. Lead uranyl acetate binds to exposed phosphate on the bone surface (e.g. hydroxyapatite crystals) [45]. Recent studies have demonstrated that fluorescent dyes used to stain microdamage do not stain all regions of permanent deformation within bone tissue [46], [47], [48]. Hence, it is possible that there are small forms of microdamage that are not stained by lead uranyl acetate. Nevertheless, in a study where high resolution imaging (order of 10 nm voxel size) was performed, lead uranyl acetate staining was typically localized to areas of maximum stress [17], suggesting lead uranyl acetate binding occurred in the regions where microdamage would be expected. Second, our staining protocol is expected to stain microdamage caused by fatigue loading as well as any pre-existing microdamage that had been caused *in vivo*. The use of multiple specimens from each donor mediated the effect of pre-existing damage on our results, as can be seen by the fact that in all but one donor increasing amounts of fatigue loading were associated with increased amounts of stained microdamage. Third, a limitation of our study was that the maximum applied strain (3500 µε) was greater than is common during activities *in vivo*. Lifting an 8 kg weight has been estimated to induce an *in vivo* apparent strain of 2300 µε in vertebral cancellous bone [49]. Thus, an apparent strain of 3500 µε is representative of an overload, but is still considerably lower than required for failure [50]. The maximum strain level was selected to balance the goals of attaining observable microdamage while limiting the amount of degradation of the material from environmental conditions (temperature, etc.) during loading [19], [51], [52].

The current study provides a detailed description of the accumulation of microdamage during fatigue loading in human vertebral cancellous bone, making it possible to estimate the biomechanical importance of microdamage, including that presumably generated *in vivo* in donor tissue [7], [53]. Determination of the relation between microdamage accumulation and reductions in Young’s modulus is not entirely new in itself; others have described such relations in bovine cancellous bone or cortical bone, but the current study is the first to determine the relation for human cancellous bone. Consistent with the linear relation between damage volume fraction and reduction in Young’s modulus reported here, Moore and colleagues found that the number of damaged trabeculae per total section area increased linearly with increasing modulus reduction in bovine cancellous bone [30].

Our measures of microdamage are of similar magnitude as previous studies. In the current study, the damage volume fraction ranged between 0.3% and 7.4%. In bovine cancellous bone subjected to an overload the damage volume fraction was 4.54±2.94% [15] and the damaged area fraction was 3%±1.9% [24]. Similar ranges in microdamage are achieved in our laboratory using serial milling or two-dimensional sections. Nevertheless, our measures of microdamage are much smaller than *in vivo* microdamage reported from analysis of basic fuchsin, where the percent bone area with diffuse damage reached values up to 50% [53]. Differences in damage volume fraction could arise from the fact that in the current study microdamage was induced by mechanical loading, which may differ from microdamage sustained *in vivo*. In the current study, microdamage was induced by cyclic compressive loading, while *in vivo* there is more likely a mixed loading mode including bending and torsion that might cause differences in the amount of microdamage. Also, differences in the population of the subjects investigated with the current study only containing older donors (range 62–92 years) and the previous study including younger donors (range 23–96 years) could lead to differences in damage volume fraction. Furthermore, staining methodology (lead uranyl acetate v. basic fuchsin), sectioning (three-dimensional v. two-dimensional sections) or measurement (thresholding v. point counts) could have contributed to the differences in the magnitudes of microdamage. The damage volume fraction in the current study was also lower than that generated in vitro and visualized using lead uranyl acetate [16], [54], where DV/BV was as high as 25%. Differences in skeletal region examined could contribute to the observed differences in microdamage. Vertebral cancellous bone is more rod-like and porous than bone from the femoral neck or tibial plateau (used by the Vashishth laboratory). Failure mechanisms of cancellous bone have been shown to differ between plate-like and rod-like bone [33], with tissue predominantly yielding in plates [55]. Therefore, more microdamage might accumulate in tibial or femoral cancellous bone before failure than in vertebral cancellous bone. There are also possible differences between damage generated from uniaxial loading (previous studies) and from fatigue loading (the current study). In addition, other factors including the lead uranyl acetate staining protocol (immersion time, rinsing methodology), image resolution, micro-CT settings (energy and integration time) and thresholding (manual v. automated thresholding with Otsu) may contribute to the differences in DV/BV between our study and studies of the Vashishth laboratory. To examine the effect of thresholding we analyzed the specimens with manually selected thresholds and found a similar range in DV/BV to that determined with the Otsu method (0.3–7.4% manually v. 0.9–8.8% with Otsu) and found that global thresholds necessary to achieve DV/BV measures as high as 25% did not match user observations, suggesting that global threshold selection alone is less likely to explain the differences in magnitude of DV/BV between our study and prior work. Further study is necessary to identify the true reason(s) for the differences.

In agreement with previous studies that have clearly shown that creep contributes to fatigue behavior in cancellous bone [28], [29], [56], creep strain dominated the accumulation of strain. Whether creep strain contributed to microdamage accumulation was, however, less clear since DV/BV and creep strain were correlated (**Fig. S1**) and *in vivo* loading models in animals have shown that creep loading causes microdamage [57], [58]. Nevertheless, others have proposed that the contribution of creep strain on damage accumulation is negligible [29], [52], [59], because most of the creep strain is recoverable upon unloading and can therefore only cause minor permanent deformation to cancellous bone [56]. Maximum strain was the single best predictor of DV/BV (tested using multivariate linear regressions), which is consistent with a strain-based failure criterion for cancellous bone [30], [42], [60], however, because creep strain and maximum strain were highly correlated it is not possible to conclude which was causative.

Consistent with previous studies that found no effect of gender on fatigue properties in bone [32], [61], the current study did not detect differences in fatigue properties between male and female samples.

In contrast to others that have reported correlations between the bone microstructure and bone microdamage [54], [62], [63], [64], in the current study DV/BV did not correlate with the bone microarchitecture, and the bone microarchitecture did not appear to influence the relationship between DV/BV and modulus reduction. In the current study BV/TV ranges from 5.5% to 15.0% while prior examinations of microdamage in cancellous bone have ranged from 17.6% to 38.4% [64]. Thus the current study may not have had a sufficient range in microarchitecture to detect a correlation between microdamage and microarchitecture.

Although the current study did not examine the bone ultrastructure, it is useful to consider the mechanisms of energy dissipation that can explain changes in mechanical properties with fatigue loading. Bone is a hierarchical structure with energy dissipation mechanisms at different length scales of bone [65]. To resist fracture, bone tissue can deform through fibrillar sliding or through the formation of microcracks [66], [67], where larger microcracks dissipate greater amounts of energy. Our observation of a correlation between DV/BV and energy dissipation per cycle are consistent with the idea that microdamage contributes to energy dissipation in cancellous bone. We speculate that in the secondary phase of fatigue loading crack-limiting boundaries (such as cement lines) limited the size of microdamage and therefore the amount of energy dissipation, while in the tertiary phase, microcracks extended through boundaries and result in greater microcracks with greater levels of energy dissipation.

Our study provides a quantitative estimation of the relationship between DV/BV and proportion of fatigue life. Our findings suggest that small amounts of microdamage in human vertebral cancellous bone cause reductions in fatigue life that are much greater than reductions in Young’s modulus. Therefore, even small amounts of stained microdamage may indicate large reductions in the number of loading cycles that the tissue can sustain before failure even when alterations in cancellous bone stiffness are small. Although the current study examined only the effects of microdamage generated by cyclic compressive loading, we expect our estimations of the effects of microdamage on subsequent biomechanical performance to be conservative because alterations in loading mode and mixed mode loading have been shown to be more effective at propagating microdamage [26], [64]. Recognizing the limitations of our study mentioned above, the current study provides a means of estimating the biomechanical importance of *in vivo* microdamage observed within cancellous bone tissue.

In conclusion, we have demonstrated the relation between microscopically observed microdamage, reductions in specimen stiffness and reduced fatigue life during cyclic loading of human vertebral cancellous bone. Our findings suggest that microdamage has a much greater effect on fatigue life than on Young’s modulus and provide a means to estimate the biomechanical repercussions of *in vivo* microdamage observed in tissue from patients.

## Supporting Information

Figure S1 **Scatterplots.** Scatterplots of correlations between DV/BV, mechanical properties, BV/TV and trabecular thickness are shown.
(TIF)
