# Supplementary figures and images for: Microdamage Caused by Fatigue Loading in Human Cancellous Bone: Relationship to Reductions in Bone Biomechanical Performance
Source: PLoS One. 2013 Dec 30;8(12):e83662. doi: 10.1371/journal.pone.0083662 (PMC3875472; doi:10.1371/journal.pone.0083662)

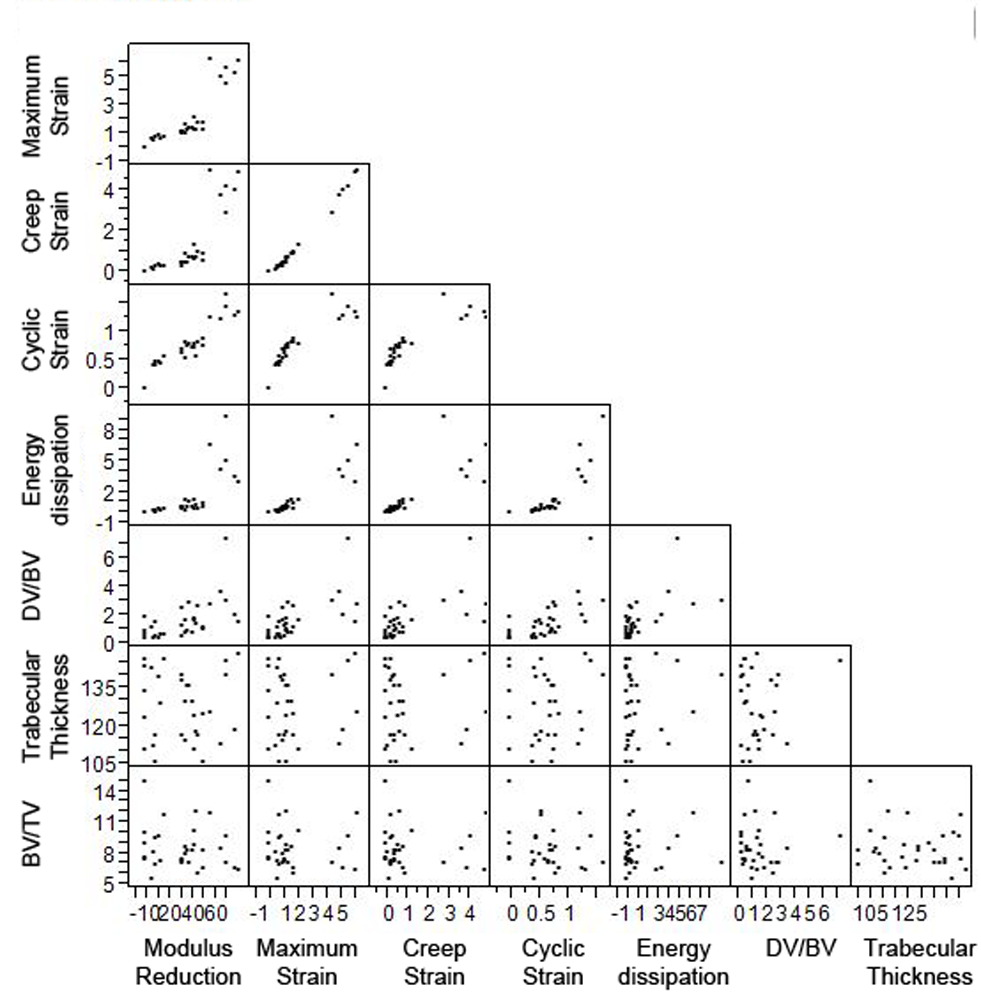

Supplement: Figure S1 — Scatterplots. Scatterplots of correlations between DV/BV, mechanical properties, BV/TV and trabecular thickness are shown. (TIF) [file pone.0083662.s001.tif]
